# The Week's Work

**Published:** 1909-11-20

**Authors:** 


### The Week's Work.

### THE CHAIRMAN KNIGHT.

Although the Birthday Honours list does not contain the name of any notable member of the profession, the knighthood conferred on Sir Frederick Macmillan, Chairman of the National Hospital for the Paralysed, will be generally welcomed. A chairman of a great voluntary hospital, who devotes much of his time to the administration of its affairs, renders an important service to his day and generation. Such services too seldom receive public recognition. Sir Frederick Macmillan was educated at Uppingham, and is chairman of the great publishing house which bears his name. He has been president of the Publishers' Association of Great Britain and Ireland, is a member of the Council of the Royal Literary Fund, and a trustee of the Booksellers' Provident Institution. He has taken a deep interest in the affairs of the National Hospital for the Paralysed for several years.

### A MATRON AND A BOARD OF GUARDIANS.

An interesting situation has arisen at the North Evington Infirmary, where the matron, called upon by the Board of Guardians to resign, has stuck to her position and refused to accept her dismissal. The Board, it will be remembered, passed a resolution calling upon the matron to resign in consequence of certain charges. To this resolution she replied denying the charges and declaring that no opportunity had been given her for refuting them, however damaging to her reputation. At its last meeting the Board had this reply in front of it, and discussed the situation thus created at some length. It was finally agreed that no action should be taken, but that the matron should be "suspended"—that is remain at her post, without authority or salary—until the Local Government Board has inquired into the matter. It is imperative that the matron should be given the opportunity she asks for to answer the charges made against her. The action of the Board in summarily dismissing her without the option of an explanation is much to be regretted, since it has put the Guardians in the unenviable position of suspending their own resolution.

### THE HOSPITALS OF CANADA.

At the meeting of the Canadian Medical Association, which was held in Winnipeg some two months ago, Dr. Bruce Smith read a paper on the Hospitals of Canada and their Relationship to the People. In the discussion that followed Dr. J. R. Jones, of Winnipeg, said that the one thing to be avoided in Canadian hospital management was the pauperisation of patients, and no movement should be tolerated which had in any way this effect. Indiscriminate charity was a most harmful influence in a community, and Europe and England were pointed out as examples of this kind of influence. In Winnipeg Englishmen often expected, as a right, to be treated free at the hospitals, an impression due largely to the practice customary in the hospitals of Great Britain. In Canada and the United States a certain amount of free medical treatment is given, but not nearly to the same extent as in Great Britain. In New York City, for instance, there is only one hospital in which patients are admitted absolutely without charge. At all the other institutions of the kind payment is expected in almost every case. It is argued that this procedure is best for all concerned. The medical men are paid for their trouble and for the expenditure of skill, while the patients preserve their self-respect and independence. The hospitals of New York are abused by persons who can well afford to pay large fees; but they are not so greatly abused as are the hospitals of the Old Country. In all the large cities of the United States, as a rule, the in-patients pay for treatment, and in Canada the tendency is to adopt as patterns in this respect the hospitals of the United States rather than those of Great Britain. Dr. Jones is quite right when he declaims against indiscriminate charity. Granted that the conditions of the people in Great Britain and Canada are different, it is wise teaching to warn against any form of pauperisation in Canada.

### WORKING MEN AND HOSPITALS.

Labouring men are showing themselves good friends of the hospitals, not only in this country, but in America and on the Continent. How excellently they respond to an appeal on behalf of their suffering fellow-men is evidenced whenever there is an annual meeting; for it is now a commonplace when the report announces that contributions from working men's societies are on the increase. A striking example is furnished by the response to the appeal recently issued on behalf of the East Suffolk and Ipswich Hospital. This response indeed has been remarkable, and should be a proof, if any were wanted, to institutional secretaries and committees of what may be done by appealing to the sympathies of organised labour. The working men of one firm alone will contribute £400 annually towards the Ipswich Hospital; another batch of working men have voluntarily doubled their subscriptions, while the women employees of another firm have taxed themselves at the rate of a halfpenny a week each to support the hospital. It is interesting to note that this cordial response has been due to the efforts of the men themselves. Labouring men have canvassed on behalf of the appeal and interested their fellow-employees in the charity. We congratulate the East Suffolk and Ipswich Hospital on possessing such staunch and practical supporters and on the quick success that has attended their appeal for funds.

### CHICHESTER GENERAL INFIRMARY.

At the annual meeting of this institution, the Governors had before them the report published in our columns on October 9. In that report it was recommended that the Governors should take active steps to raise the £2,000 which will more than discharge its outstanding liabilities. At the annual meeting held on the 11th inst. this suggestion was fully and sympathetically discussed. Major Jellicourse, in an able speech, referring to the King's regret, declared it was a golden opportunity for the infirmary to go a-begging. He strongly urged that if properly worked an appeal would produce all the money needed. Prebendary Fraser, Mr. W. D. James, and others supported this view, and on the motion of Mr. James it was decided to appoint a Finance Sub-Committee to consider what course should be taken to improve the finances of the infirmary, with power to take such steps as they may think best to produce this result. The committee consists of the Bishop of Chichester, the Mayor of Chichester, Major Jellicourse, Chancellor Davey, Mr. James, and Alderman Gibbins. The Bishop has proved himself to be a giant in raising money for philanthropic purposes; the Mayor should be glad of the opportunity now afforded him to show that municipal authority in Chichester is a living force, whilst Major Jellicourse and Mr. James, who exhibited the right spirit in their speeches, will no doubt bring the necessary interest and energy to bear, and so speedily secure the £2,000. We wish the movement all success. We look forward to the pleasure of congratulating the Governors of this old foundation, which should soon be known as the Royal West Sussex, East Hants, and Chichester Hospital.

### WORK AND PAY.

A somewhat cryptic correspondence has taken place between Mr. Godfrey Hamilton, Secretary of the National Hospital for the Paralysed, and Mr. B. Burford Rawlings, its former secretary. Mr. Burford Rawlings having described hospital secretaries as men whose energies are industriously directed to an unselfish pursuit of gold, and who burn with a lust of acquisition into which enters no thought of personal gain, whilst they neither participate in honours nor share the rewards of successful enterprise, Mr. Hamilton raised his voice in protest. Mr. Hamilton very properly urged that hospital secretaries, like other people, if they can raise money successfully are rewarded, and hope for increased recognition. Indeed, men who are training for the post of secretary to a hospital find that experience and success in raising money are very often important considerations with the committees making such appointments. Mr. Rawlings, in reply, urged that there are not a few men whose pride and pleasure it is to return something more than an equivalent for salary received. He instances workers to whom the prosperity of their institution is a coveted prize dear beyond words, though it may bring no sort of material reward to themselves. Mr. Burford Rawlings rightly urges that men of this spirit in the office of secretary are essential to the prosperity of every voluntary hospital of importance. We are inclined to sympathise with both contestants, knowing that if a man will put himself into his work, and do it always to the utmost of his ability, the work will take care of the man. It is a moot point whether hospital secretaries are paid adequately or not. We are inclined to think that many of them are not, holding that a great hospital is a business, and, like all important businesses, those who control its management should be adequately and well paid for their work. If all voluntary hospitals were endowed institutions we might incline to the feeling that good pay might result in somnolence rather than work. There is no fear, however, that the secretary of an important voluntary hospital in these days will ever laugh and grow fat, for the work he has to do is far too strenuous, if he retains his post, to render any such catastrophe possible. On the other hand, no good man is justified in continuing to work for a great hospital if he is not adequately paid for his services. Roughly, we should say that a hospital superintendent and secretary's salary should amount to something like 5 per cent. of its total voluntary income. Our experience is that much retarded justice might be done if some hospital committees would look into this question of secretaries' salaries and deal with it on its merits.

### THE ELECTION OF THE MEDICAL STAFF.

A very interesting question has been engaging public attention in Victoria in regard to the mode of election of the medical staff of the Melbourne Hospital. It has been the custom there to appoint members of the honorary staff for four years, and an election which is vested in the whole body of Governors is held once in four years. This plan has given rise to dissatisfaction and even abuse, with the result that a special committee, composed of the members of the Hospital Committee, of the University Council, of the Faculty of Medicine in the University, and of the honorary staff, was appointed to bring up new rules regulating such elections. These have been formulated and are now under discussion. They provide that each member of the honorary staff shall hold office for six years, but shall retire in any case at sixty years of age. Each physician and surgeon and medical officer in charge of a special department will eventually hold office for six years and no longer, but will be eligible for re-election. The period of office is so regulated that one physician and one surgeon to in-patients and one physician and one surgeon to out-patients will retire each year. None of the existing staff will be called upon to retire until the expiry of their present term of office. An Advisory Board or committee of election is set up, consisting of ten members, four appointed by the Managing Committee, two by the medical staff, two by the Council of Melbourne University, and two by the Faculty of Medicine; all such members to be chosen annually. All appointments are vested in the Advisory Board at their discretion, who have the power to pass over the seniors and to recommend other members of the staff for appointment when a vacancy occurs, but this discretion will not be operative unless it is exercised by an absolute majority of the Board. Power is given to the Committee of Management by an absolute majority to suspend any member of the medical staff from office, subject to appeal to the whole body of Governors. It was explained by the Rev. E. S. Hughes, who moved the alteration of the rules, that they had been very difficult to draft, but the honorary staff as a whole had accepted them. The plan we have just explained "was not ideal, but it was at least practicable." We agree with Mr. Hughes in this opinion, and shall watch the working of the new rules with considerable interest.

